# A Clinicopathology Review and Update of Epstein–Barr Virus-Associated Mesenchymal Tumors

**DOI:** 10.3390/cancers15235563

**Published:** 2023-11-24

**Authors:** Oswald Zhao Jian Lee, Noorjehan Omar, Joshua K. Tay, Victor Kwan Min Lee

**Affiliations:** 1 Department of Pathology, National University Hospital, National University Health System, Singapore 119074, Singapore; oswald.lee@mohh.com.sg; 2 Department of Pathology, Hospital Serdang, Kajang 43000, Malaysia; noorjehan.omar@gmail.com; 3 Department of Otolaryngology—Head and Neck Surgery, National University of Singapore, Singapore 119228, Singapore; 4 Department of Pathology, Yong Loo Lin School of Medicine, National University of Singapore, Singapore 119228, Singapore

**Keywords:** Epstein–Barr virus (EBV), mesenchymal, sarcoma, smooth muscle

## Abstract

**Simple Summary:**

Despite an in-depth understanding of Epstein–Barr virus-associated epithelial and lymphoid tumors, there is a paucity of knowledge concerning mesenchymal tumors associated with the Epstein–Barr virus. Our objective with this review article is to provide a comprehensive reference, offering current insights into the clinical features, pathological characteristics, pathophysiology, prognostic factors, and current therapeutic approaches of Epstein–Barr virus-associated mesenchymal tumors.

**Abstract:**

The Epstein–Barr virus (EBV) is associated with various tumor types, including nasopharyngeal carcinoma and lymphoproliferative disorders. While much is known about EBV-related epithelial and lymphoid tumors, there is a paucity of knowledge concerning EBV-associated mesenchymal tumors. This review aims to provide a comprehensive overview of EBV-associated mesenchymal tumors, encompassing their clinical features, pathological characteristics, pathophysiology, prognostic factors, and current treatment approaches. Through an extensive literature search using the PubMed database, we were able to identify three distinct EBV-associated mesenchymal tumors: EBV-associated smooth muscle tumors, inflammatory pseudotumor-like follicular dendritic cell sarcomas, and EBV-associated osteosarcomas. Although this review extensively explored the different aspects of these mesenchymal tumors, our comprehension of the underlying pathophysiology in this context is still incomplete. Therefore, we hope that this review paper will not only serve as a valuable repository of information but also serve as a catalyst for prospective in vitro and in vivo research studies to bridge the existing knowledge gap surrounding pathophysiology, ultimately making an important contribution to shaping future therapeutic approaches.

## 1. Introduction

Approximately 15–20% of human malignancies are caused by seven major oncogenic viruses [1,2,3]. These viruses belong to two categories: DNA viruses (such as the Epstein–Barr virus (EBV), Kaposi sarcoma-associated herpesvirus (KSHV), Hepatitis B virus (HBV), human papillomaviruses (HPV), and Merkel cell polyomavirus (MCPyV)) and RNA viruses (including the human T-lymphotropic virus 1 (HTLV-1) and Hepatitis C virus (HCV)). EBV falls within the γ subfamily of herpesviruses, a group of lymphotropic viruses that also includes the KSHV, herpesvirus saimiri, and murine herpesvirus [4]. In the 1950s, Denis Burkitt discovered Burkitt’s lymphoma in East Africa and proposed a connection between mosquito-transmitted viruses and human cancers, intriguing researchers [5,6]. In 1965, Anthony Epstein and colleagues established cell lines from Burkitt’s lymphomas, finding herpes virus-like particles [7,8]. Werner and Gertrude Henle later confirmed this virus as distinct from other human herpesviruses, naming it EBV [9].

EBV is ubiquitous in nature and can infect approximately 95% of the human population via exposure to oral secretions. In most cases, this infection does not exhibit any noticeable symptoms. However, when it affects adolescents and young adults, it can give rise to a condition known as infectious mononucleosis [10]. EBV, a linear, double-stranded DNA virus, contains over 100 genes, but only a specific subset plays a role in the viral life cycle. These include non-coding RNAs (EBER 1, EBER2, microRNAs), nuclear antigens (EBNA1, EBNA2, EBNA3A, EBNA3B, EBNA3C, LP), and latent membrane proteins (LMP1, LMP2A, and LMP2B). These subsets of genes are expressed in various combinations and are categorized into three main latency programs:

- Latency 0: Antigen-negative infection.
- Latency I: Expression of EBNA1.
- Latency II: Expression of EBNA1, LMP1, LMP2A, and LMP2B.
- Latency I/II: Expression patterns between latency I and II.
- Latency III: Expression of all EBNA proteins, microRNAs, as well as LMP1, LMP2A, and LMP2B.

**Figure 1:**
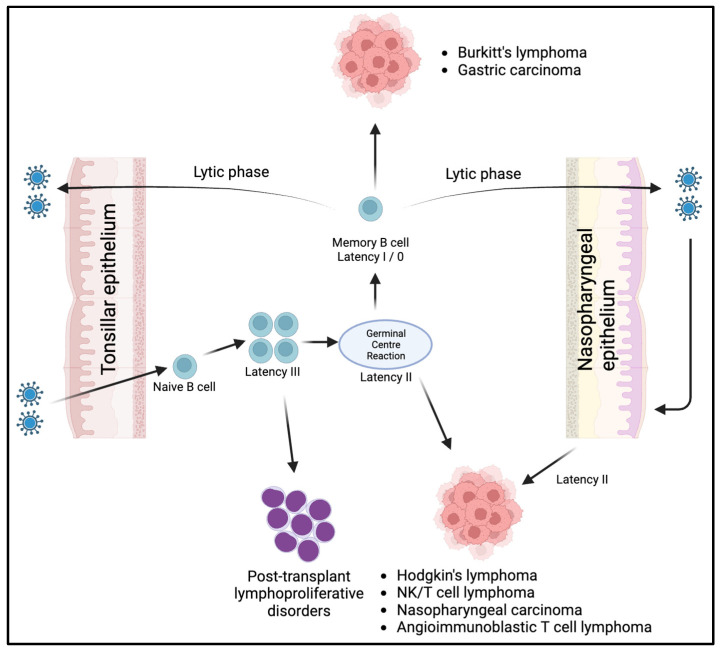
Illustration of the life cycle of EBV and its associated latency programs, leading to the development of different types of tumors.

As illustrated in Figure 1, during the initial infection, the orally transmitted virus enters dormant B cells by binding to the B cell complement receptor (CD21) via the EBV glycoprotein gp350/220. Subsequently, another viral glycoprotein, gp42, binds to HLA class II molecules, leading to the internalization of the virus. Following infection, the virus typically adopts a latency III program within naive B cells located in the oropharyngeal lymphoid tissues. This program allows the infected cells to proliferate within these tissues. The B cells transformed by the virus then decrease the expression of viral antigens (latency I/0), cease their proliferation, and circulate in the bloodstream as memory B cells. These memory B cells function as the main reservoir of latent infection in EBV carriers. The exact mechanism responsible for this transformation into memory B cells remains unclear. One possible pathway involves a germinal center (GC) reaction, while an alternative route could be a non-GC pathway. The reactivation of these memory B cells into the lytic cycle, potentially triggered with plasma cell differentiation, leads to the production of virions. Subsequently, these virions can initiate new latent B cell infections, establish new sites of virus replication in distant epithelial surfaces, or continuously reinfect the oropharyngeal epithelium [11,12,13]. This theory finds support in the fact that the virus has been discovered in various locations far from the oropharynx, such as breast milk, cervical epithelium, and semen, implying the possible transportation of the virus by B cells to these sites [14,15,16]. Moreover, when EBV-targeted treatment is discontinued, the virus can be detected in oropharyngeal secretions at levels comparable to those before treatment [17].

Latent EBV genes have also been implicated in the tumorigenesis via inhibiting apoptosis and enabling immune evasion [18]. A study by Lajoie et al. demonstrated that LMP1 induced a downregulation of the shelterin proteins, such as TRF2. These proteins are important for DNA replication and protecting chromosome ends. When the shelterin expression levels are reduced, cytokinesis is disrupted, leading to the observation of multinucleation in Reed–Sternberg cells in EBV-associated Hodgkin’s lymphoma [19]. Figure 1 illustrates the connection between EBV latency programs and EBV-related malignancies. Immunodeficiency-associated lymphoproliferative disorders such as post-transplant lymphoproliferative diseases, exhibit latency III. Hodgkin’s lymphoma, NK/T cell lymphomas, angioimmunoblastic T cell lymphoma, and nasopharyngeal carcinoma exhibit latency II. Gastric carcinoma and Burkitt lymphoma exhibit latency I. Latency 0, characterized by its dormant state, is not known to be associated with malignancies [12]. Despite the fact that the majority of the world’s population is infected with EBV, most individuals do not develop EBV-associated malignancies. This has led to the suggestion that other factors also play a significant role in tumor development. For instance, Burkitt’s lymphoma cells not only harbor the EBV genome but also undergo a chromosomal translocation, causing aberrant overexpression of the MYC oncogene. In a study where B cells were transformed with EBV in vitro, it was revealed that there was an initial low cloning capacity and an inability to form tumors in mice. However, after the introduction of the altered MYC oncogene, these EBV-transformed cells exhibited enhanced cellular growth [20]. These findings imply that viruses often serve as initiators in the carcinogenic process, supporting the idea that cancer development results from the accumulation of multiple events rather than a single event.

Despite having a thorough understanding of cancers in epithelial and lymphoid tissues associated with the Epstein–Barr virus (EBV), there is limited knowledge about EBV-associated mesenchymal tumors. Through an extensive search of the PubMed database, we were able to identify three specific types of EBV-associated mesenchymal tumors: EBV-associated smooth muscle tumors (EBV-SMTs), inflammatory pseudotumor-like follicular dendritic cell sarcomas (IPT-FDCSs), and EBV-associated osteosarcomas. The main objective of this review is to broaden and deepen our knowledge about these mesenchymal tumors. This encompasses an in-depth analysis of their clinical features, pathological characteristics, pathophysiology, prognostic factors, and the current treatment methods available.

## 2. EBV-Associated Smooth Muscle Tumors

Over the recent five decades, EBV-SMTs have been documented in individuals with compromised immune systems. The first reports of malignant smooth muscle tumors in immunosuppressed patients date back to the 1970s when Pritzker et al. made the initial observations. In the 1990s, the link between EBV and these malignant smooth muscle tumors was established, leading to their characterization as EBV-SMTs [21,22]. 

According to the available literature (see Table S1), EBV-SMT represents a subgroup of smooth muscle tumors occurring in individuals with weakened immune systems. This condition can manifest at any age, ranging from 1.5 to 63 years, and is predominantly found in female patients with uncontrolled HIV, those undergoing immunosuppressive treatment after organ transplantation, or individuals with congenital immunodeficiency disorders. EBV-SMTs can affect virtually any organ, including visceral sites, the central nervous system, spine, or peripheral soft tissues [22,23,24,25,26,27,28,29,30,31,32,33,34,35,36,37,38,39,40,41,42,43,44,45,46,47,48,49,50,51,52,53,54,55,56,57,58]. These tumors can appear at single or multiple sites either simultaneously or at different times but do not exhibit metastatic behavior. A study by Deyrup et al. examined EBV-SMTs in 19 patients with multiorgan involvement and found that, in each case, the tumors were genetically distinct, suggesting that the multiorgan condition arises from multiple primary tumors rather than metastasis [59]. Moreover, it has been observed that EBV-SMTs related to HIV infection tend to have a higher likelihood of central nervous system involvement [25,26,27,29,31,35,43,45,52,59].

**Figure 2:**
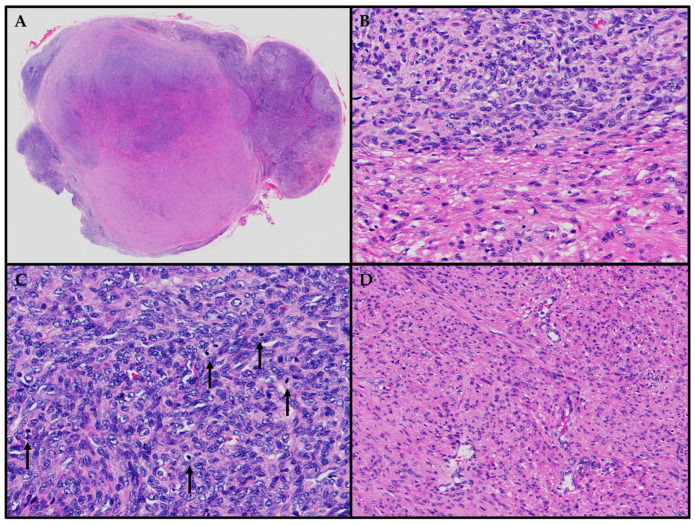
Hematoxylin and eosin stain. (**A**) A lymph node involved with a well-circumscribed EBV-SMT. (**B**) Two separate groups of tumor cells, with one resembling a typical leiomyoma, while the second group exhibits increased nuclear atypia with a higher nuclear atypia to the cytoplasmic (NC) ratio. (**C**) There is a focally increased number of mitotic figures, particularly within the group of tumor cells with a higher NC ratio (indicated by black arrows). (**D**) Increased vascularity is evident, characterized by the presence of delicate, thin-walled blood vessels within the tumor.

Histologically, as detailed in Table S1, EBV-SMTs share several characteristics with typical leiomyomas. These features include well-defined borders and fascicles of spindle-shaped cells with “cigar-shaped” nuclei andmoderate amount of eosinophilic cytoplasm [22,23,24,25,26,27,28,29,30,31,32,33,34,35,36,37,38,39,40,41,42,43,44,45,46,47,48,49,50,51,52,53,54,55,56,57,58]. In some cases, as reported by Suwan et al., these tumor cells may exhibit epithelioid morphology [49]. Notably, there can be focal areas within EBV-SMTs that display increased nuclear atypia, necrosis, or increased mitotic activity, with up to 20 mitotic figures per 10 high-power fields and a Ki67 proliferative index of up to 24% [23,24,32,35,36,42,43,45,47,51,52,53]. The background stroma can occasionally show collagenous or myxoid changes [23,29,45]. The distinctive characteristics of EBV-SMTs comprise the presence of a second population of small, round tumor cells with hyperchromatic, round nuclei and limited amphophilic cytoplasm, as well as an infiltrate of intratumoral lymphocytes [26,28,29,30,34,36,41,44,45,47,48,50,51,53,55,57]. Other noteworthy features may include increased vascularity resembling hemangiopericytoma (HPC)-like vasculature or an appearance reminiscent of an inflammatory pseudotumor (IPT) [27,28,29,34,53]. Visual representations of the key histological features described earlier are also presented in an illustrative case of a lymph node involved with an EBV-SMT (see Figure 2).

In one case described by Debiec et al., an EBV-SMT exhibited two separate areas: one with a conventional smooth muscle component and another with an IPT-like component. Interestingly, molecular analysis revealed two related aberrant clones, one with an ALK gene loss and the other with a translocation of the 3′ end of the ALK kinase domain on the der (3) chromosome, corresponding to the smooth muscle and IPT components, respectively. Despite these molecular differences, both areas displayed the same cytogenetic alterations, suggesting a single clonal population. Notably, ALK activation could not be demonstrated in the IPT component [28]. Another case report by Sprangers et al. also did not detect any ALK gene rearrangement with FISH in an EBV-SMT with IPT-like morphology [48]. Thus, these findings could indicate that the tumorigenesis of EBV-SMT might involve other EBV-induced mechanisms, apart from the direct ALK activation. 

**Figure 3:**
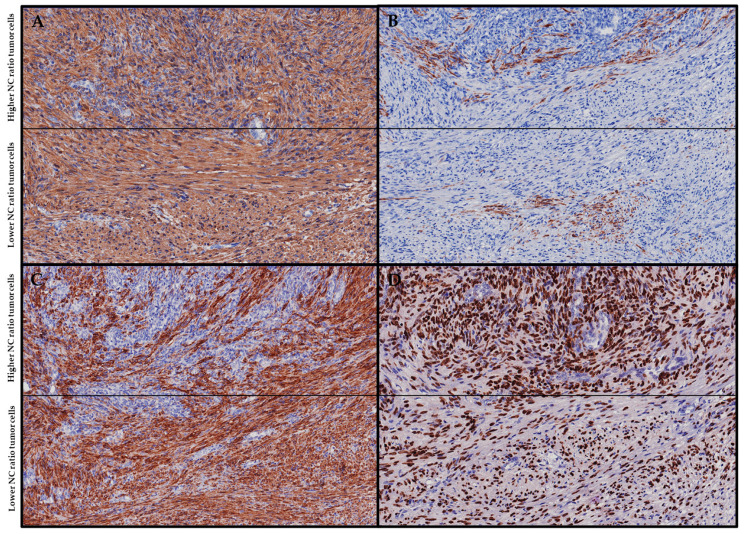
Immunohistochemistry. (**A**) Both tumor populations, exhibiting both higher and lower NC ratios, demonstrate diffuse SMA positivity. (**B**) Both tumor populations display patchy desmin positivity. (**C**) Patchy h-caldesmon positivity is observed within the tumor population with a higher NC ratio, whereas the tumor population with a lower NC ratio shows diffuse h-caldesmon positivity. (**D**) Both tumor populations display diffuse EBER-ish positivity.

EBV-SMTs are typically positive for smooth muscle markers, such as smooth muscle actin (SMA), desmin, and h-caldesmon. In situ hybridization for EBV-encoded RNA (EBER-ish) is also typically positive, while CD21 staining may show variable positivity [22,23,24,25,26,27,28,29,30,31,32,33,34,35,36,37,38,39,40,41,42,43,44,45,46,47,48,49,50,51,52,53,54,55,56,57,58]. Visual representations of the key immunohistochemical features described earlier are also presented in an illustrative case of a lymph node involved with an EBV-SMT (see Figure 3). Due to the diverse morphological patterns present in EBV-SMTs, several differential diagnoses should be considered. Fortunately, these differentials can be effectively ruled out by a combination of immunohistochemical and special histochemical stains, as summarized in Table 1. Moreover, thorough sampling of the tumor mass to reveal classical areas can be instrumental in confirming an EBV-SMT diagnosis, especially when only a primitive round cell component is observed.

Various explanations for the mechanism of EBV entry into the precursor cells of these tumors have been proposed. The histological examination of EBV-SMTs in relation to blood vessels raises the possibility of a viral preference for vascular smooth muscle. Some researchers have demonstrated that in certain HIV-positive individuals with EBV-SMTs, the presence of CD21, a receptor that EBV can bind to, allows for an entry of EBV into smooth muscle cells. This could result in a latent infection and a subsequent development of tumors. Interestingly, there are also cases of post-organ transplant EBV-SMTs where CD21 is absent. Given the presence of intratumoral lymphocytes within the EBV-SMT, an alternative theory has been suggested, involving a fusion event between smooth muscle cells and an infected B-lymphocyte occurring prior to tumor proliferation [60,61,62]. According to Wah et al., the gene set enrichment analysis demonstrated a copy number gain of genes involved in the host response to viral infection in EBV-SMTs, suggesting that the tumor immune microenvironment could have initiated infected cell transformation and tumor growth [63]. However, this fusion hypothesis has also been challenged by a study conducted by Soares et al. using immunohistochemistry, in which they found that the majority of intratumoral lymphocytes were CD3+ and CD8+, and none of these lymphocytes are positive for EBER-ish. This suggests that these lymphocytes are likely reactive and may not have a significant role in the entry of EBV into host cells [47]. After entering host cells, a potential molecular mechanism underlying EBV-SMT tumorigenesis appears to involve the reactivation of the Akt/mammalian target of rapamycin (mTOR) signaling pathway triggered by EBV-LMP2A. This reactivation leads to the overexpression of the MCY, which in turn promotes cellular proliferation and the formation of tumors [36,64,65,66,67].

In the most recent WHO classification from 2020, SMTs have been categorized into different groups: benign tumors, such as leiomyomas (LMs); intermediate tumors, including EBV-SMTs and SMTs of uncertain malignant potential; and malignant tumors, which encompass leiomyosarcomas (LMSs) [68]. The classification of EBV-SMTs as intermediate tumors is due to the uncertainty surrounding their biological behavior. For instance, the histological grading of EBV-SMTs does not serve as a reliable prognostic indicator. A comprehensive review by Purgina et al. analyzed 64 published cases of EBV-SMTs and found no difference in clinical outcomes between those labeled as “SMT” and “LMSs” [62]. Nevertheless, certain adverse clinical features, such as the involvement of multiple organs and intracranial regions, have been demonstrated to correlate with decreased overall survival in EBV-SMTs [30,35,36,40,42,55]. A recent genomic study conducted by Wah et al. revealed that EBV-SMTs exhibit a similar burden of copy number alterations as leiomyomas and lower than leiomyosarcomas. This molecular distinction aligns with the differences in the clinical behavior observed between EBV-SMTs, LMs, and LMSs. Although EBV-SMTs cluster more closely with leiomyomas in terms of genomics, the tumors also display a copy number gain of oncogenic proteins, such as RUNX1, CCND2, and ETS2, indicating a potential malignant potential within these tumors [63]. Due to the uncertainty regarding the biological behavior of EBV-SMTs, many experts suggest avoiding the term “LMSs” and instead recommend using the term “SMTs”.

Although there are currently no universally accepted standard treatment protocols for EBV-SMTs, several approaches have been suggested:

- Surgical resection: Surgical removal of tumors remains one of the primary treatment options, particularly for patients with unifocal diseases. Notably, there are no reports of disease recurrences in patients who have undergone surgical resections [32,34,42,47,49,51,56]. While challenging for multifocal diseases, surgical resections can still be considered, especially when the tumor is symptomatic or involves intracranial structures. Complete surgical removal in such cases may improve survival rates [58,69].
- Immune reconstitution: Given the multifocal nature of EBV-SMT and its association with immunodeficiency, numerous reports propose prioritizing immune system restoration as a fundamental treatment approach. For patients with HIV, it is recommended to utilize antiretroviral therapy to reinstate immune function [27,35]. In the case of organ transplant recipients, reducing immunosuppressive medications is advised to bolster the immune response [36,39,41,48]. In individuals with congenital immunodeficiency disorders, the option of a bone marrow transplant may be contemplated as a means to enhance immune function [44,55].
- mTOR inhibitors: Given the pivotal role of the mTOR pathway in tumorigenesis, there is an interest in using mTOR inhibitors, such as sirolimus, for treating EBV-SMT. Some reports indicate disease control and regression following sirolimus use [41,66,70,71].
- Other treatment modalities: Chemotherapy and radiotherapy are occasionally used; however, they are generally considered ineffective in treating these tumors [36]. Combining EBV-directed antiviral therapy to suppress EBV replication may enhance disease control and potentially lead to better outcomes [48,72,73]. Additionally, a novel treatment approach has been explored for patients with post-transplant smooth muscle tumors (PTSMT). This involves the adoptive cell transfer (ACT) of EBV-specific T cells. One case study reported a stable disease and reduced EBV viremia in a 20-year-old patient who had undergone cardiac transplantation and developed PTSMT [33].

## 3. Inflammatory Pseudotumor-like Follicular Dendritic Cell Sarcoma

IPT-FDCS is a unique tumor associated with EBV infection, as opposed to other inflammatory pseudotumors (IPTs). The concept of IPTs was first described in 1903 and encompassed various conditions, including infective, autoimmune, or reactive fibroinflammatory processes [74,75,76,77,78]. Inflammatory myofibroblastic tumors (IMTs), on the other hand, were distinguished from this heterogeneous group of IPTs and are classified as intermediate-grade neoplasms, largely due to the identification of recurrent gene rearrangements [78]. It is important to note that some older reports may have inaccurately categorized splenic and liver IPT-FDCSs as IMTs or other IPTs. Similarly, some splenic and liver EBV-SMTs may have been mistakenly reported as IPT-FDCSs, EBV-associated IPTs, or EBV-associated IMTs. This misclassification could have occurred due to the nonspecific nature of SMA positivity within the tumor, which is known to come from various cellular origins, including fibroblastic, myofibroblastic, fibrohistiocytic, fibroblastic reticular cells, as well as follicular dendritic and smooth muscle cells [75,79,80,81,82].

FDCS, a rare entity included in the 2017 ‘WHO Classification of Tumors’, was initially described in 1986 as an uncommon mesenchymal neoplasm arising from the FDCs found in lymphoid tissues [83,84,85]. This tumor is categorized into two subtypes: conventional FDCS and IPT-FDCS. IPT-FDCS was first identified in 2001 and represents a distinct subtype of FDCS that consistently exhibits EBV positivity [75,86]. FDCS is known to affect individuals across a wide age range, spanning from 7 to 82 years old, with a prevalence among middle-aged adults [85,87,88,89]. When comparing the two subtypes, EBV-related IPT-FDCS typically occurs in older age groups, with an average age of 60.7 years, while the non-EBV-related conventional FDCS tends to affect slightly younger individuals, with an average age of 45.3 years [90]. IPT-FDCS also demonstrates a slight female predominance, with a ratio of 2.2:1, whereas the non-EBV-related conventional FDCS exhibits male predominance, with a ratio of 3:2 [80,87,88,89,91]. The majority of IPT-FDCS cases are reported in the liver and spleen, with some exceptional cases documented in lymph nodes, the gastrointestinal tract, and the lungs [75,79,80,89,92]. In contrast, conventional FDCS can occur in both nodal and extranodal sites, manifesting at a wide range of locations, including the oral cavity, soft palate, and tonsils [83,84,85,87].

**Figure 4:**
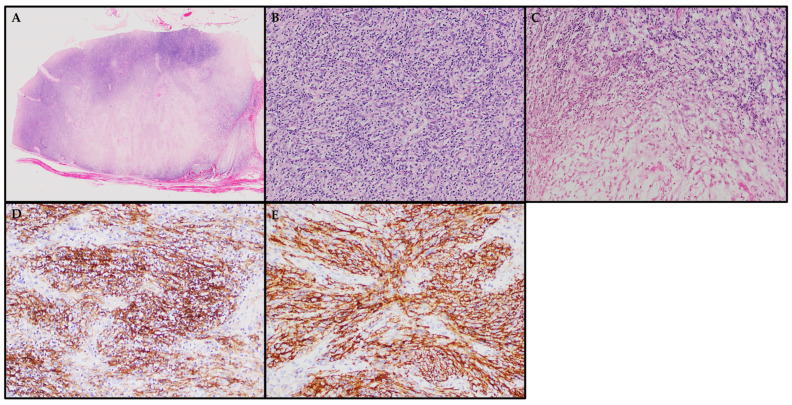
Hematoxylin and eosin stain and immunohistochemistry. (**A**) A lymph node involved with a well-circumscribed IPT-FDCS comprising two distinct hypercellular and hypocellular areas. (**B**) Hypercellular areas comprising fascicles of spindle cells with admixed dense lymphocytic infiltrates. (**C**) The juxtaposition of hypercellular areas with hypocellular myxoid areas. (**D**,**E**) Neoplastic spindle cells from a different FDCS case displaying positive expression of CD21 and CD35, respectively.

All cases of IPT-FDCS exhibit common histological features, including spindle-shaped cells arranged in various patterns, such as fascicles, whorls, or storiform patterns. These FDCs display an IPT-like morphology characterized by a dense infiltration of T-lymphocytes, which distinguishes them from conventional FDCS, where only scattered lymphocytes are typically present [75,80,88,93,94]. Perivascular lymphocyte cuffs and a mixed population of eosinophils, plasma cells, and neutrophils are also frequently seen. Mitotic figures are usually rare, and some cases may contain foci of necrosis, hemorrhage, noncaseating epithelioid granulomas, or intravascular fibrin clots. The cytomorphology of IPT-FDCS include oval to elongated nuclei with dispersed chromatin, indistinct nucleoli, fibrillary eosinophilic cytoplasm with syncytial cellular borders. Occasionally, binucleate or multinucleate cells and cells with nuclear pseudoinclusions can be observed [85]. Visual representations of the key histological features described earlier are also presented in an illustrative case of a lymph node involved with an IPT-FDCS (see Figure 4A–C).

Immunohistochemical staining plays a crucial role in confirming the diagnosis of IPT-FDCS. These tumors consistently show positivity for typical FDC markers, such as CD21, CD35, CD23, clusterin, podoplanin, CXCL13, and CAN.4, which helps to differentiate them from other entities (see Figure 4D,E) [80,85,88,93,95,96]. Some authors have also reported positivity for newer markers, such as follicular dendritic cell secreted protein (FDCSP) and serglycin (SRGN), through whole transcriptome sequencing. Variable staining intensity has also been noted for claudin4, CD68, and fascin [97,98]. It is important to note that older case reports documented positivity for SMA, which is now understood to potentially represent what is known as EBV-SMT and IMT. TdT staining highlights the presence of immature T cell lymphoblastic proliferation within the tumor in approximately 45% of cases. When abundant, this finding may be associated with paraneoplastic autoimmune multiorgan syndrome [99]. EBER-ish is positive in 92.1% of IPT-FDCS cases, and EBV-LMP1 is seen in 90% of cases, compared to conventional FDCS—where these markers are typically absent [85,88]. While a number of cases have exhibited EBER-ish positivity in lymphocytes that are positive for CD3 and CD20, many experts maintain the belief that these instances could potentially be attributable to bystander EBV-infected lymphocytes or the presence of a concurrent lymphoproliferative disorder. Double-labeling techniques provide additional support for the notion that these EBV markers are primarily detected within the follicular dendritic cells themselves, rather than solely within the adjacent bystander EBV-infected lymphocytes in the background. Given the wide range of potential differential diagnoses for IPT-FDCS, a panel of immunohistochemical stains is essential to accurately discriminate between them (see Table 2).

While conventional FDCSs have been linked to follicular dendritic cell proliferation in immune disorders, such as the hyaline vascular variant of Castleman disease, the underlying tumorigenesis in IPT-FDCS remains largely unknown. In many other EBV-related epithelial, lymphoid, and mesenchymal neoplasms, the development of tumors is linked to immunosuppressive conditions, such as uncontrolled HIV, iatrogenic immunosuppression in transplant patients, and congenital immunocompromised states. In such instances, the suppression of the immune system can trigger the activation of EBV latency-associated genes, ultimately resulting in the development of tumors. However, this mechanism has not been observed in any of the previously reported cases of IPT-FDCS [80,86,88,89,91,100,101]. This aspect represents an active area of ongoing research and investigation, and further studies are needed to unravel the underlying mechanisms contributing to the development of IPT-FDCS.

Contrary to conventional FDCS, which frequently recurs and potentially metastasize, IPT-FDCS typically shows an indolent clinical course [102]. As per the study conducted by Wu et al., the majority of patients have achieved successful treatment outcomes through a surgical intervention alone. Only a small fraction of cases (15.8%) has experienced local recurrences or distant metastases after their initial treatment. However, analytical investigations, which include Kaplan–Meier and multivariate analyses, have failed to establish a significant correlation between factors such as patient age, gender, tumor size, and morphological characteristics with regard to disease prognosis [91]. The aforementioned findings align with our literature review findings. In most instances of IPT-FDCS, favorable outcomes have been observed following the surgical removal of the tumor. Recurrences have been infrequent, and instances of metastasis have been documented in only a very small number of cases [75,80,85,87,88,90,92].

Surgical excision is considered the treatment of choice for IPT-FDCS. Given its typically indolent behavior, the role of adjuvant therapy remains unclear when it comes to managing this type of tumor [86,91]. However, due to the possibility of a recurrence, regular surveillance is recommended [86].

## 4. EBV-Associated Osteosarcoma

The existing literature on the association between EBV and osteosarcoma is limited, with only one reported retrospective cohort study conducted by Mardanpour et al. This study explored the presence of EBV in osteosarcoma and its potential impact on prognosis [103].

This study included 48 immunocompetent patients, all of whom were Iranian, with a mean age of 17.43 years. Among these patients, 33 (69%) were boys, and 15 (31%) were girls, resulting in a male-to-female ratio of 2.2:1 [103]. All cases exhibited histological characteristics consistent with a conventional osteosarcoma. This study also found that only 6 out of the 48 osteosarcoma cases exhibited a co-expression of EBV-encoded small RNA1 (EBER1) and latent membrane protein 1 (LMP1) in tumor cells [103].

In all cases, surgical tumor removal was performed without the administration of neoadjuvant chemotherapy or radiation therapy. Importantly, the authors observed that the simultaneous presence of EBER1 and LMP1 was linked to unfavorable clinical characteristics, including lymph node metastasis, distant metastasis, and an advanced tumor stage. Over an average follow-up period of 45 ± 12 months, this study found that individuals who tested positive for EBER1 and LMP1 exhibited significantly poorer outcomes compared to those who tested negative for these markers. Specifically, the 5-year overall survival rate was 54% for patients with EBER1 and LMP1 positivity, in contrast to 85% for those who were negative for these markers. Likewise, the 5-year progression-free survival rate was 41% for patients with EBER1 and LMP1 positivity, as opposed to 81% for those without these markers. Furthermore, patients with EBER1 and LMP1 positivity experienced considerably shorter disease-free survival periods compared to their counterparts who tested negative for these markers [103].

While this study provided valuable insights into the prognostic significance of EBV markers in osteosarcoma patients, the underlying mechanisms contributing to the development of osteosarcoma in the context of EBV infection were not addressed. Interestingly, in this study on EBV-associated osteosarcomas, the focus was primarily on immunocompetent patients, revealing a higher occurrence among males. Conversely, other studies on EBV-associated soft tissue tumors, such as EBV-SMTs, centered on immunodeficient patients, showing a higher frequency among females. These findings suggest that, in addition to the specific tumor types, both immune status and demographic factors could influence the biological behaviors of these tumors. However, it is important to acknowledge that the conclusions derived from Mardanpour et al.’s study are derived from a limited sample size and are confined to a specific geographic region [103]. Therefore, further studies with larger sample sizes and diverse geographic locations are necessary to validate these findings.

## 5. Conclusions and Future Directions

While our review paper offers a comprehensive overview and presents the latest insights into rare mesenchymal tumors, our knowledge of the underlying mechanisms driving tumorigenesis in these cases is still limited. This lack of understanding of the pathophysiology explains the absence of standardized treatment guidelines for these tumors. Several key questions in the pathophysiology of these tumors warrant further exploration:

- Identifying specific EBV strains in mesenchymal tumors: Recent genomic studies have revealed diverse EBV strains with specific affinities for different lymphoid and epithelial cells [104,105]. Despite this, the identification of EBV strains capable of transforming mesenchymal tissues has proven elusive. Investigating these specific strains in mesenchymal tumors could provide valuable insights into the virus’s genetic diversity.
- Decoding viral entry mechanisms into mesenchymal cells: Various hypotheses have been proposed about how EBV infects host tissues, including the involvement of CD21 receptors. The theory suggesting a CD21-mediated entry as the mechanism for EBV infection is supported by the presence of CD21 in B cells and non-lymphoid cells, including mesenchymal cells [106,107]. However, the exact viral entry mechanism into mesenchymal cells remains unknown due to the challenges in establishing mesenchymal tissue cell lines for in vitro studies. Therefore, efforts should focus on developing techniques to culture mesenchymal cell line tissues, enabling comprehensive research on how EBV binds to these cells.
- Identifying potential biomarkers in tumor signaling pathways: Although the molecular mechanisms in EBV-associated epithelial or lymphoid neoplasms are well comprehended, there remains a gap in the knowledge concerning how EBV induces tumorigenesis in mesenchymal tissues. The importance of comprehending these disease processes is exemplified by the finding of the mTOR signaling pathway’s involvement in EBV-SMT. This discovery has led to the utilization of mTOR inhibitors, such as sirolimus, demonstrating their effectiveness in treating EBV-SMT patients [41,66,70,71]. Ongoing research efforts are thus essential in identifying potential biomarkers within tumor signaling pathways, offering promising targets for therapy in these groups of patients.

Apart from the lack of understanding of the pathophysiology of Epstein–Barr virus-associated mesenchymal tumors, the absence of randomized controlled trials involving patients with these mesenchymal tumors further complicates the establishment of standardized protocols. Until standardized treatment strategies are established, it is essential to note that the choice of treatment should be tailored to the individual patient’s condition, including the extent of the disease, overall health, and the presence of an underlying immunodeficiency, and should be made in consultation with a multidisciplinary medical team.

Our objective with this review article was to provide a comprehensive reference, offering current insights into the various aspects of EBV-associated mesenchymal tumors. However, it is important to note that our current understanding of pathophysiology in this context remains incomplete. Therefore, we hope that this review paper will not only serve as a valuable repository of information but also serve as a catalyst for prospective in vitro and in vivo research studies to bridge the existing knowledge gap, ultimately making a substantial contribution to shaping future therapeutic approaches.

## Figures and Tables

**Table 1:** Illustration of the different immuno- and special histochemical stains to distinguish EBV-SMT from other possible differential diagnoses.

Tumors	Cytokeratins	SMA	Desmin	H-Caldesmon	S100	HMB45	CD21	CD23	CD34	CD35	CD68	CD117	DOG1	ALK1	EBER-Ish	HHV-8	Ziehl-Neelsen
EBV-SMT	−	+	+	+	−	−	+/−	−	−	−	−	−	−	−	+	−	−
**Differentials of EBV**-**SMT with Spindled Morphology**																	
Non-EBV SMT	−	+	+	+	−	−	−	−	−	−	−	−	−	−	−	−	−
Spindle Cell GIST	−	+	−	−	−	−	−	−	+	−	−	+	+	−	−	−	−
Peripheral Nerve Sheath Tumors	−	−	−	−	+	−	−	−	−	−	−	−	−	−	−	−	−
Kaposi Sarcoma	−	−	−	−	−	−	−	−	+	+	−	−	−	−	−	+	−
Mycobacterial Spindle Cell Pseudotumor	−	−	−	−	+	−	−	−	−	−	+	−	−	−	−	−	+
**Differentials of EBV**-**SMT with Epithelioid Morphology**																	
Carcinoma	+	−	−	−	−	−	−	−	−	−	−	−	−	−	−	−	−
Melanoma	−	−	−	−	+	+	−	−	−	−	−	−	−	−	−	−	−
Epithelioid GIST	−	+	−	−	−	−	−	−	+	−	−	+	+	−	−	−	−
Epithelioid Sarcoma	+	−	−	−	−	−	−	−	+	−	−	−	−	−	−	−	−
**Differentials of EBV**-**SMT with Increased Vascularity/HPC**-**like Morphology**																	
Myopericytoma	−	+	−	+	−	−	−	−	−	−	−	−	−	−	−	−	−
Angioleiomyoma	−	+	+	+	−	−	−	−	−	−	−	−	−	−	−	−	−
**Differentials of EBV**-**SMT with IPT**-**like Morphology**																	
IMT	−	+	−	−	−	−	−	−	−	−	−	−	−	+	−	−	−
IPT-FDCS	−	+	−	−	−	−	+	+	−	+	−	−	−	−	+	−	−

GIST = Gastrointestinal Stromal Tumor; IMT = Inflammatory Myofibroblastic Tumor.

**Table 2:** Illustration of the different immunohistochemical stains to distinguish IPT-FDCS from other possible differential diagnoses.

Tumors	Cytokeratins	SMA	Desmin	H-Caldesmon	S100	HMB45	CD21	CD23	CD34	CD35	CD68	CD163	CD1a	ALK1	EBER-Ish	HHV-8
IPT-FDCS	−	+	−	−	−	−	+	+	−	+	+	−	−	−	+	−
EBV-SMT	−	+	+	+	−	−	+/−	−	−	−	−	−	−	−	+	−
IMT	−	+	−	−	−	−	−	−	−	−	−	−	−	+	−	−
IDCS	−	−	−	−	+	−	−	−	−	−	+	+	−	−	−	−
Kaposi Sarcoma	−	−	−	−	−	−	−	−	+	−	−	−	−	−	−	+
LCH	−	−	−	−	+	−	−	−	−	−	+	+	+	−	−	−
Carcinoma	+	−	−	−	−	−	−	−	−	−	−	−	−	−	−	−
Melanoma	−	−	−	−	+	+	−	−	−	−	−	−	−	−	−	−

IDCS = Interdigitating dendritic cell sarcoma; LCH = Langerhans Cell Histiocytosis.

## Supplementary Materials

The following supporting information can be downloaded at: https://www.mdpi.com/article/10.3390/cancers15235563/s1, Table S1: Summary of EBV-SMT cases.

## References

[1] zur Hausen H., de Villiers E.-M. (2014). Cancer “Causation” by Infections—Individual Contributions and Synergistic Networks. Semin. Oncol..

[2] Varn F.S., Schaafsma E., Wang Y., Cheng C. (2018). Genomic Characterization of Six Virus-Associated Cancers Identifies Changes in the Tumor Immune Microenvironment and Altered Genetic Programs. Cancer Res..

[3] Kitsou K., Iliopoulou M., Spoulou V., Lagiou P., Magiorkinis G. (2021). Viral Causality of Human Cancer and Potential Roles of Human Endogenous Retroviruses in the Multi-Omics Era: An Evolutionary Epidemiology Review. Front. Oncol..

[4] Deyrup A.T. (2008). Epstein-Barr Virus–Associated Epithelial and Mesenchymal Neoplasms. Hum. Pathol..

[5] Burkitt D. (1962). A Children’s Cancer Dependent on Climatic Factors. Nature.

[6] Levine A.J. (1991). Viruses New York.

[7] Epstein M.A., Henle G., Achong B.G., Barr Y.M. (1965). Morphological and biological studies on a virus in cultured lymphoblasts from burkitt’s lymphoma. J. Exp. Med..

[8] Epstein M.A., Achong B.G., Barr Y.M. (1964). Virus particles in cultured lymphoblasts from burkitt’s lymphoma. Lancet.

[9] Henle G., Henle W. (1966). Immunofluorescence in Cells Derived from Burkitt’s Lymphoma. J. Bacteriol..

[10] Cohen J.I. (2015). Primary Immunodeficiencies Associated with EBV Disease. Curr. Top. Microbiol. Immunol..

[11] Shannon-Lowe C., Rickinson A. (2019). The Global Landscape of EBV-Associated Tumors. Front. Oncol..

[12] Chakravorty S., Afzali B., Kazemian M. (2022). EBV-Associated Diseases: Current Therapeutics and Emerging Technologies. Front. Immunol..

[13] Baumforth K.R., Young L.S., Flavell K.J., Constandinou C., Murray P.G. (1999). The Epstein-Barr Virus and Its Association with Human Cancers. Mol. Pathol..

[14] Israele V., Shirley P., Sixbey J.W. (1991). Excretion of the Epstein-Barr Virus from the Genital Tract of Men. J. Infect. Dis..

[15] Sixbey J.W., Lemon S.M., Pagano J.S. (1986). A Second Site for Epstein-Barr Virus Shedding: The Uterine Cervix. Lancet.

[16] Junker A.K., Thomas E.E., Radcliffe A., Forsyth R.B., Davidson A.G., Rymo L. (1991). Epstein-Barr Virus Shedding in Breast Milk. Am. J. Med. Sci..

[17] Yao Q.Y., Ogan P., Rowe M., Wood M., Rickinson A.B. (1989). Epstein-Barr Virus-Infected B Cells Persist in the Circulation of Acyclovir-Treated Virus Carriers. Int. J. Cancer.

[18] Thorley-Lawson D.A., Gross A. (2004). Persistence of the Epstein–Barr Virus and the Origins of Associated Lymphomas. N. Engl. J. Med..

[19] Lajoie V., Lemieux B., Sawan B., Lichtensztejn D., Lichtensztejn Z., Wellinger R., Mai S., Knecht H. (2015). LMP1 Mediates Multinuclearity through Downregulation of Shelterin Proteins and Formation of Telomeric Aggregates. Blood.

[20] Lombardi L., Newcomb E.W., Dalla-Favera R. (1987). Pathogenesis of Burkitt Lymphoma: Expression of an Activated c-Myc Oncogene Causes the Tumorigenic Conversion of EBV-Infected Human B Lymphoblasts. Cell.

[21] Liebowitz D. (1995). Epstein-Barr Virus—An Old Dog with New Tricks. N. Engl. J. Med..

[22] McClain K.L., Leach C.T., Jenson H.B., Joshi V.V., Pollock B.H., Parmley R.T., DiCarlo F.J., Chadwick E.G., Murphy S.B. (1995). Association of Epstein-Barr Virus with Leiomyosarcomas in Young People with AIDS. N. Engl. J. Med..

[23] Al-Tarawneh H., Alp A., Gedikoglu G., Kosemehmetoglu K. (2023). Epstein-Barr Virus-Positive Leiomyosarcoma in Immunocompetent Patients. Turk Patoloji Derg..

[24] Anbardar M.H., Soleimani N., Safavi D., Eshraghian A., Ayoub A. (2022). Multifocal EBV-Associated Smooth Muscle Tumors in a Patient with Cytomegalovirus Infection after Liver Transplantation: A Case Report from Shiraz, Iran. Diagn. Pathol..

[25] Barrett K., Tavakoli S., McGinity M., Gilbert A. (2022). Intracranial Epstein-Barr Virus-Associated Smooth Muscle Tumor with Superimposed Cryptococcal Infection: A Case Report. Medicine.

[26] Chiu Y.-T., Lee J.-C., Cheng A., Hsieh S.-M. (2018). Epstein-Barr Virus-Associated Smooth Muscle Tumor as the Initial Presentation of HIV Infection: A Case Report. J. Formos. Med. Assoc..

[27] Chong Y.B., Lu P.-L., Ma Y.-C., Yin H.-L., Chang C.-H. (2022). Epstein-Barr Virus-Associated Smooth Muscle Tumor and Its Correlation With CD4 Levels in a Patient With HIV Infection. Front. Cell. Infect. Microbiol..

[28] Debiec-Rychter M., Croes R., De Vos R., Marynen P., Roskams T., Hagemeijer A., Lombaerts R., Sciot R. (2003). Complex Genomic Rearrangement of ALK Loci Associated with Integrated Human Epstein-Barr Virus in a Post-Transplant Myogenic Liver Tumor. Am. J. Pathol..

[29] Ehresman J.S., Ahmed A.K., Palsgrove D.N., Pennington Z., Goodwin C.R., Sciubba D.M. (2018). Epstein-Barr Virus-Associated Smooth Muscle Tumor Involving the Spine of an HIV-Infected Patient: Case Report and Review of the Literature. J. Clin. Neurosci..

[30] Fournier B., Hoshino A., Bruneau J., Bachelet C., Fusaro M., Klifa R., Lévy R., Lenoir C., Soudais C., Picard C. (2022). Inherited TNFSF9 Deficiency Causes Broad Epstein-Barr Virus Infection with EBV+ Smooth Muscle Tumors. J. Exp. Med..

[31] Galeano-Piedrahita E., Rico A.M.M., Suárez A.C.R., Walter A.L. (2021). Cutaneous Smooth Muscle Tumors Associated with Epstein-Barr Virus in an Adult Patient with HIV. An. Bras. Dermatol..

[32] Hamed M.O., Roberts K.J., Merchant W., Lodge J.P.A. (2015). Contemporary Management and Classification of Hepatic Leiomyosarcoma. HPB.

[33] Hansen B.-T., Bacher P., Eiz-Vesper B., Heckl S.M., Klapper W., Koch K., Maecker-Kolhoff B., Baldus C.D., Fransecky L. (2021). Adoptive Cell Transfer of Allogeneic Epstein-Barr Virus-Specific T Lymphocytes for Treatment of Refractory EBV-Associated Posttransplant Smooth Muscle Tumors: A Case Report. Front. Immunol..

[34] Huang J., Loh K.S., Petersson F. (2010). Epstein-Barr Virus-Associated Smooth Muscle Tumor of the Larynx: Report of a Rare Case Mimicking Leiomyosarcoma. Head Neck Pathol..

[35] Issarachaikul R., Shuangshoti S., Suankratay C. (2014). Epstein-Barr Virus-Associated Smooth Muscle Tumors in AIDS Patients: A Largest Case (Series). Intern. Med..

[36] Jonigk D., Laenger F., Maegel L., Izykowski N., Rische J., Tiede C., Klein C., Maecker-Kolhoff B., Kreipe H., Hussein K. (2012). Molecular and Clinicopathological Analysis of Epstein-Barr Virus-Associated Posttransplant Smooth Muscle Tumors. Am. J. Transplant..

[37] Johnson B.M., Iskandar J.-P., Farha N., Yerian L., Modaresi Esfeh J., Lindenmeyer C. (2022). Hepatic Epstein-Barr Virus-Associated Smooth Muscle Tumor in a Heart and Liver Transplant Recipient. ACG Case Rep. J..

[38] Kang Z., Xu J., Li Z. (2021). Juvenile Idiopathic Arthritis With Epstein-Barr Virus-Associated Smooth Muscle Tumor in a 6-Year-Old Girl: A Rare Case Report. Front. Pediatr..

[39] Khan A.A., Estfan B.N., Yalamanchali A., Niang D., Savage E.C., Fulmer C.G., Gosnell H.L., Modaresi Esfeh J. (2022). Epstein-Barr Virus-Associated Smooth Muscle Tumors in Immunocompromised Patients: Six Case Reports. World J. Clin. Oncol..

[40] Lee E.S., Locker J., Nalesnik M., Reyes J., Jaffe R., Alashari M., Nour B., Tzakis A., Dickman P.S. (1995). The Association of Epstein-Barr Virus with Smooth-Muscle Tumors Occurring after Organ Transplantation. N. Engl. J. Med..

[41] Liu Y., Chintalapati S., Dietz R., Raza A.S., Wang J., Raza A.S. (2017). EBV-Associated Hepatic Smooth Muscle Tumor of Uncertain Biologic Behavior after Heart Transplantation in a Pediatric Patient: Case Report. J. Gastrointest. Oncol..

[42] Moore Dalal K., Antonescu C.R., Dematteo R.P., Maki R.G. (2008). EBV-Associated Smooth Muscle Neoplasms: Solid Tumors Arising in the Presence of Immunosuppression and Autoimmune Diseases. Sarcoma.

[43] Munjal S., Warade A., Samantray S., Madiwale C., Desai K. (2017). Dorsolumbar Spine Epstein Barr Virus Associated Leiomyosarcoma in a Human Immunodeficiency Virus Patient. Neurol. India.

[44] Parta M., Cuellar-Rodriguez J., Freeman A.F., Gea-Banacloche J., Holland S.M., Hickstein D.D. (2017). Resolution of Multifocal Epstein-Barr Virus-Related Smooth Muscle Tumor in a Patient with GATA2 Deficiency Following Hematopoietic Stem Cell Transplantation. J. Clin. Immunol..

[45] Pitjadi T.M., Grayson W. (2019). Epstein-Barr Virus-Associated Smooth Muscle Tumour: A Case Series with a Significant Proportion of Tumours Showing Proclivity for Cutaneous Soft Tissues. Dermatopathology.

[46] Shi Q., Tang W.-F., He X.-L., Tian X. (2021). Epstein-Barr virus-associated smooth muscle tumor in a girl. Zhongguo Dang Dai Er Ke Za Zhi.

[47] Soares C.D., Carlos R., Molina J.P.D., de Lima Morais T.M., de Almeida O.P. (2019). Laryngeal Epstein-Barr Virus-Associated Smooth Muscle Tumor in an Undernourished Child. Head Neck Pathol..

[48] Sprangers B., Smets S., Sagaert X., Wozniak A., Wollants E., Van Ranst M., Debiec-Rychter M., Sciot R., Vanrenterghem Y., Kuypers D.R. (2008). Posttransplant Epstein-Barr Virus-Associated Myogenic Tumors: Case Report and Review of the Literature. Am. J. Transplant..

[49] Suwan Y., Rojanaporn D., Teekhasaenee C., Keelawat S. (2016). Epstein-Barr Virus-Associated Iris Smooth Muscle Tumor with Epithelioid Morphology in AIDS Patients: A Case Report. Int. Med. Case Rep. J..

[50] Tabor J.K., Lei H., Morales-Valero S.F., O’Brien J., Gopal P.P., Erson-Omay E.Z., Fulbright R.K., Moliterno J. (2023). Epstein-Barr Virus-Associated Primary Intracranial Leiomyosarcoma in an Immunocompetent Patient: Illustrative Case. J. Neurosurg. Case Lessons.

[51] Whaley R.D., Thompson L.D.R. (2021). Epstein-Barr Virus-Associated Smooth Muscle Tumors of Larynx: A Clinicopathologic Study and Comprehensive Literature Review of 12 Cases. Head Neck Pathol..

[52] Wilaisakditipakorn T., Vilaisaktipakorn P., Bunupuradah T., Puthanakit T. (2015). A Slow Progressor HIV-Infected Boy Developing Quadriplegia with Evidence of Epstein-Barr Virus Associated Smooth Muscle Tumour of the Cervical Spinal Cord. BMJ Case Rep..

[53] Wood D., Matsika A., Srinivasan B., Watson R. (2018). Epstein Barr Virus-Associated Smooth Muscle Tumour (EBV-SMT) of the Urinary Bladder. Urol. Case Rep..

[54] Yokoyama D., Horiguchi K., Higuchi Y., Hashiba J. (2020). Transnasal Endoscopic Resection of Epstein-Barr Virus-Associated Cavernous Sinus Tumour. BMJ Case Rep..

[55] Yonkof J.R., Gupta A., Rueda C.M., Mangray S., Prince B.T., Rangarajan H.G., Alshahrani M., Varga E., Cripe T.P., Abraham R.S. (2020). A Novel Pathogenic Variant in CARMIL2 (RLTPR) Causing CARMIL2 Deficiency and EBV-Associated Smooth Muscle Tumors. Front. Immunol..

[56] Yu L., Aldave A.J., Glasgow B.J. (2009). Epstein-Barr Virus-Associated Smooth Muscle Tumor of the Iris in a Patient with Transplant: A Case Report and Review of the Literature. Arch. Pathol. Lab. Med..

[57] Yuan H., Zeng B., He L., Tan X., Jiang L. (2022). 18F-FDG PET/CT Imaging of Multiple Intrahepatic Epstein-Barr Virus-Associated Smooth Muscle Tumors in a Pediatric Patient after Heart Transplantation. Hell. J. Nucl. Med..

[58] Zhou Q., Wu F., Guo Y., Zhu B. (2020). Epstein-Barr Virus Associated Hepatic Smooth Muscle Tumor in a Patient with Acquired Immunodeficiency Syndrome: A Case Report. Medicine.

[59] Deyrup A.T., Lee V.K., Hill C.E., Cheuk W., Toh H.C., Kesavan S., Chan E.W., Weiss S.W. (2006). Epstein-Barr Virus-Associated Smooth Muscle Tumors Are Distinctive Mesenchymal Tumors Reflecting Multiple Infection Events: A Clinicopathologic and Molecular Analysis of 29 Tumors from 19 Patients. Am. J. Surg. Pathol..

[60] Tetzlaff M.T., Nosek C., Kovarik C.L. (2011). Epstein-Barr Virus-Associated Leiomyosarcoma with Cutaneous Involvement in an African Child with Human Immunodeficiency Virus: A Case Report and Review of the Literature. J. Cutan. Pathol..

[61] Adam E., Wang L., Herrington C., Bliss D., Church J.A. (2014). Synchronous HIV/AIDS-Related Epstein-Barr Virus-Associated Smooth Muscle Tumors in a 20-Year-Old Female. Pediatr. Infect. Dis. J..

[62] Purgina B., Rao U.N.M., Miettinen M., Pantanowitz L. (2011). AIDS-Related EBV-Associated Smooth Muscle Tumors: A Review of 64 Published Cases. Patholog. Res. Int..

[63] Wah N.W., Mok Y., Omar N., Chang K.T.E., Tay T.K.Y., Hue S.S.-S., Lee V.K.M. (2023). Clinicopathologic and Molecular Characteristics of Epstein-Barr Virus-Associated Smooth Muscle Tumor Compared with Those of Leiomyoma and Leiomyosarcoma. Mod. Pathol..

[64] Jenson H.B., Montalvo E.A., McClain K.L., Ench Y., Heard P., Christy B.A., Dewalt-Hagan P.J., Moyer M.P. (1999). Characterization of Natural Epstein-Barr Virus Infection and Replication in Smooth Muscle Cells from a Leiomyosarcoma. J. Med. Virol..

[65] Moody C.A., Scott R.S., Amirghahari N., Nathan C.-A., Young L.S., Dawson C.W., Sixbey J.W. (2005). Modulation of the Cell Growth Regulator mTOR by Epstein-Barr Virus-Encoded LMP2A. J. Virol..

[66] Ong K.W., Teo M., Lee V., Ong D., Lee A., Tan C.S., Vathsala A., Toh H.C. (2009). Expression of EBV Latent Antigens, Mammalian Target of Rapamycin, and Tumor Suppression Genes in EBV-Positive Smooth Muscle Tumors: Clinical and Therapeutic Implications. Clin. Cancer Res..

[67] Shen Q., Feng W., Long M.S., Duan X., Jaijakul S., Arias C.A., Brown R.E., Zhao B. (2011). Multicentric Hepatic EBV-Associated Smooth Muscle Tumors in an AIDS Patient: A Case Report, Investigation of mTOR Activation and Review of the Literature. Int. J. Clin. Exp. Pathol..

[68] Choi J.H., Ro J.Y. (2021). The 2020 WHO Classification of Tumors of Soft Tissue: Selected Changes and New Entities. Adv. Anat. Pathol..

[69] Lau K.-W., Hsu Y.-W., Lin Y.-T., Chen K.-T. (2021). Role of Surgery in Treating Epstein-Barr Virus-Associated Smooth Muscle Tumor (EBV-SMT) with Central Nervous System Invasion: A Systemic Review from 1997 to 2019. Cancer Med..

[70] Tan C.S., Loh H.-L., Foo M.W.Y., Choong L.H.L., Wong K.S., Kee T.Y.S. (2013). Epstein-Barr Virus-Associated Smooth Muscle Tumors after Kidney Transplantation: Treatment and Outcomes in a Single Center. Clin. Transplant..

[71] Toh H.C., Teo M., Ong K.W., Lee V., Chan E., Lee A.S.G., Vathsala A. (2006). Use of Sirolimus for Epstein-Barr Virus-Positive Smooth-Muscle Tumour. Lancet Oncol..

[72] Rogatsch H., Bonatti H., Menet A., Larcher C., Feichtinger H., Dirnhofer S. (2000). Epstein-Barr Virus-Associated Multicentric Leiomyosarcoma in an Adult Patient after Heart Transplantation: Case Report and Review of the Literature. Am. J. Surg. Pathol..

[73] Bonatti H., Hoefer D., Rogatsch H., Margreiter R., Larcher C., Antretter H. (2005). Successful Management of Recurrent Epstein-Barr Virus-Associated Multilocular Leiomyosarcoma after Cardiac Transplantation. Transplant. Proc..

[74] Hoebers F.J.P., Ordonez B.P., Irish J., Simpson R.E., Yu E., O’Sullivan B. (2012). Progressive Tumefactive Fibroinflammatory Lesion of the Infratemporal Fossa Treated by Radiation Therapy. Rare Tumors.

[75] Van Baeten C., Van Dorpe J. (2017). Splenic Epstein-Barr Virus-Associated Inflammatory Pseudotumor. Arch. Pathol. Lab. Med..

[76] East E.G., Carter C.S., Sciallis A.P. (2019). Cellular Spindled Histiocytic Pseudotumor: A Benign Mimic of Spindle Cell Neoplasia of the Breast. Arch. Pathol. Lab. Med..

[77] Sachdev R., Mohapatra I., Goel S., Ahlawat K., Sharma N. (2020). Core Biopsy Diagnosis of ALK Positive Inflammatory Myofibroblastic Tumor of Lung: An Interesting Case. Turk Patoloji Derg..

[78] Siemion K., Reszec-Gielazyn J., Kisluk J., Roszkowiak L., Zak J., Korzynska A. (2022). What Do We Know about Inflammatory Myofibroblastic Tumors?—A Systematic Review. Adv. Med. Sci..

[79] Arber D.A., Kamel O.W., van de Rijn M., Davis R.E., Medeiros L.J., Jaffe E.S., Weiss L.M. (1995). Frequent Presence of the Epstein-Barr Virus in Inflammatory Pseudotumor. Hum. Pathol..

[80] Morales-Vargas B., Deeb K., Peker D. (2021). Clinicopathologic and Molecular Analysis of Inflammatory Pseudotumor-Like Follicular/Fibroblastic Dendritic Cell Sarcoma: A Case Report and Review of Literature. Turk Patoloji Derg..

[81] Pagliuca F., Ronchi A., Auricchio A., Lieto E., Franco R. (2022). Inflammatory Pseudotumor-like Follicular/Fibroblastic Dendritic Cell Sarcoma: Focus on Immunohistochemical Profile and Association with Epstein-Barr Virus. Infect. Agent. Cancer.

[82] Gong S., Auer I., Duggal R., Pittaluga S., Raffeld M., Jaffe E.S. (2015). Epstein-Barr Virus-Associated Inflammatory Pseudotumor Presenting as a Colonic Mass. Hum. Pathol..

[83] Monda L., Warnke R., Rosai J. (1986). A Primary Lymph Node Malignancy with Features Suggestive of Dendritic Reticulum Cell Differentiation. A Report of 4 Cases. Am. J. Pathol..

[84] Chan Y.F., White J., Brash H. (1994). Metachronous Pulmonary and Cerebral Inflammatory Pseudotumor in a Child. Pediatr. Pathol..

[85] Wu H., Liu P., Xie X.-R., Chi J.-S., Li H., Xu C.-X. (2021). Inflammatory Pseudotumor-like Follicular Dendritic Cell Sarcoma: Literature Review of 67 Cases. World J. Metaanal..

[86] Pascariu A.D., Neagu A.I., Neagu A.V., Băjenaru A., Bețianu C.I. (2021). Hepatic Inflammatory Pseudotumor-like Follicular Dendritic Cell Tumor: A Case Report. J. Med. Case Rep..

[87] Wang H., Su Z., Hu Z., Wen J., Liu B. (2010). Follicular Dendritic Cell Sarcoma: A Report of Six Cases and a Review of the Chinese Literature. Diagn. Pathol..

[88] Ge R., Liu C., Yin X., Chen J., Zhou X., Huang C., Yu W., Shen X. (2014). Clinicopathologic Characteristics of Inflammatory Pseudotumor-like Follicular Dendritic Cell Sarcoma. Int. J. Clin. Exp. Pathol..

[89] Pan S.-T., Cheng C.-Y., Lee N.-S., Liang P.-I., Chuang S.-S. (2014). Follicular Dendritic Cell Sarcoma of the Inflammatory Pseudotumor-like Variant Presenting as a Colonic Polyp. Korean J. Pathol..

[90] Gui H., Chaudhari J., Mannan R. (2022). Follicular Dendritic Cell Sarcoma of Gastrointestinal Tract with Two Emerging Distinct Subtypes: A Case Report and Systemic Review. Diagn. Pathol..

[91] Wu C.-Y., Wang R.-C., Chen B.-J., Chen W.-Y., Jhuang J.-Y., Chang M.-C., Wu Y.-H., Nakada N., Karube K., Chuang S.-S. (2020). Granuloma with an Underlying Lymphoma: A Diagnostic Challenge and a Wider Histologic Spectrum Including Adult T-Cell Leukemia/Lymphoma. Appl. Immunohistochem. Mol. Morphol..

[92] He H., Xue Q., Tan F., Yang L., Wang X., Gao Y., Mao Y., Mu J., Wang D., Zhao J. (2021). A Rare Case of Primary Pulmonary Inflammatory Pseudotumor-like Follicular Dendritic Cell Sarcoma Successfully Treated by Lobectomy. Ann. Transl. Med..

[93] Grogg K.L., Lae M.E., Kurtin P.J. (2004). Clusterin Expression Distinguishes Follicular Dendritic Cell Tumors from Other Dendritic Cell Neoplasms: Report of a Novel Follicular Dendritic Cell Marker and Clinicopathologic Data on 12 Additional Follicular Dendritic Cell Tumors and 6 Additional Interdigitating Dendritic Cell Tumors. Am. J. Surg. Pathol..

[94] Cossu A., Lissia A., Dedola M.F., Deiana A., Faedda R., Palmieri G., Tanda F. (2005). Classic Follicular Dendritic Reticulum Cell Tumor of the Lymph Node Developing in a Patient with a Previous Inflammatory Pseudotumor–like Proliferation. Hum. Pathol..

[95] Yu H., Gibson J.A., Pinkus G.S., Hornick J.L. (2007). Podoplanin (D2-40) Is a Novel Marker for Follicular Dendritic Cell Tumors. Am. J. Clin. Pathol..

[96] Vermi W., Lonardi S., Bosisio D., Uguccioni M., Danelon G., Pileri S., Fletcher C., Sozzani S., Zorzi F., Arrigoni G. (2008). Identification of CXCL13 as a New Marker for Follicular Dendritic Cell Sarcoma. J. Pathol..

[97] Grogg K.L., Macon W.R., Kurtin P.J., Nascimento A.G. (2005). A Survey of Clusterin and Fascin Expression in Sarcomas and Spindle Cell Neoplasms: Strong Clusterin Immunostaining Is Highly Specific for Follicular Dendritic Cell Tumor. Mod. Pathol..

[98] Facchetti F., Lonardi S., Gentili F., Bercich L., Falchetti M., Tardanico R., Baronchelli C., Lucini L., Santin A., Murer B. (2007). Claudin 4 Identifies a Wide Spectrum of Epithelial Neoplasms and Represents a Very Useful Marker for Carcinoma versus Mesothelioma Diagnosis in Pleural and Peritoneal Biopsies and Effusions. Virchows Arch..

[99] Walters M., Pittelkow M.R., Hasserjian R.P., Harris N.L., Macon W.R., Kurtin P.J., Rech K.L.G. (2018). Follicular Dendritic Cell Sarcoma with Indolent T-Lymphoblastic Proliferation Is Associated With Paraneoplastic Autoimmune Multiorgan Syndrome. Am. J. Surg. Pathol..

[100] Cheuk W., Chan J.K.C., Shek T.W.H., Chang J.H., Tsou M.-H., Yuen N.W.F., Ng W.-F., Chan A.C.L., Prat J. (2001). Inflammatory Pseudotumor-Like Follicular Dendritic Cell Tumor: A Distinctive Low-Grade Malignant Intra-Abdominal Neoplasm with Consistent Epstein–Barr Virus Association. Am. J. Surg. Pathol..

[101] Lu S.S., Wang Z., Zhao S., Liu W.P. (2021). Hepatic inflammatory pseudotumor-like follicular dendritic cell sarcoma with paravertebral metastasis and recurrence: Report of a case. Zhonghua Bing Li Xue Za Zhi.

[102] Chan J.K., Fletcher C.D., Nayler S.J., Cooper K. (1997). Follicular Dendritic Cell Sarcoma. Clinicopathologic Analysis of 17 Cases Suggesting a Malignant Potential Higher than Currently Recognized. Cancer.

[103] Mardanpour K., Rahbar M., Mardanpour S., Khazaei S., Rezaei M. (2020). Co-Expression of Epstein–Barr Virus–encoded RNA1 and Viral Latent Membrane Protein 1 in Osteosarcoma: A Novel Insight of Predictive Markers. Tumour Biol..

[104] Yu F., Syn N.L., Lu Y., Chong Q.Y., Lai J., Tan W.J., Goh B.C., MacAry P.A., Wang L., Loh K.S. (2021). Characterization and Establishment of a Novel EBV Strain Simultaneously Associated With Nasopharyngeal Carcinoma and B-Cell Lymphoma. Front. Oncol..

[105] Xu M., Yao Y., Chen H., Zhang S., Cao S.-M., Zhang Z., Luo B., Liu Z., Li Z., Xiang T. (2019). Genome Sequencing Analysis Identifies Epstein-Barr Virus Subtypes Associated with High Risk of Nasopharyngeal Carcinoma. Nat. Genet..

[106] Birkenbach M., Tong X., Bradbury L.E., Tedder T.F., Kieff E. (1992). Characterization of an Epstein-Barr Virus Receptor on Human Epithelial Cells. J. Exp. Med..

[107] Timens W., Boes A., Vos H., Poppema S. (1991). Tissue Distribution of the C3d/EBV-Receptor: CD21 Monoclonal Antibodies Reactive with a Variety of Epithelial Cells, Medullary Thymocytes, and Peripheral T-Cells. Histochemistry.

